# *Klebsiella pneumoniae* liver abscess: a case report

**DOI:** 10.1186/s13256-018-1924-4

**Published:** 2018-12-13

**Authors:** P. N. S. Premathilake, W. K. S. Kularatne, J. P. K. Jayathilake, S. D. N. Senadhira

**Affiliations:** Department of Medicine, Teaching Hospital Kandy, Kandy, Sri Lanka

**Keywords:** Liver abscess, *Klebsiella pneumoniae*, Sri Lanka

## Abstract

**Background:**

Liver abscess has formerly been a polymicrobial infection. Currently, liver abscess due to *Klebsiella pneumoniae* is increasingly reported, predominantly in Southeast Asia for unknown reasons. Liver abscess due to *Klebsiella* pneumonia has never been previously reported in Sri Lanka.

**Case presentation:**

A 63-year-old Sinhalese man with diabetes mellitus and a poor glycemic control presented with fever, loose stools, and loss of appetite of 1 week’s duration. An examination was unremarkable apart from a mild non-tender hepatomegaly. Investigations indicated a septic process with neutrophil leukocytosis, thrombocytopenia, and raised inflammatory markers with acute kidney injury. Sonography of his abdomen revealed a large liver abscess with two blood cultures positive for *Klebsiella pneumoniae*. He made a complete recovery following aspiration of the abscess and treatment with intravenously administered ceftriaxone.

**Conclusion:**

Liver abscess due to *Klebsiella pneumoniae* is an emerging infection and most commonly reported from Southeast Asia. In Sri Lanka, further studies are necessary to understand the epidemiology and modes of spread. Furthermore, a high index of suspicion is essential as early detection is the key to successful treatment and prevention of complications.

## Introduction

Liver abscess due to *Klebsiella pneumoniae* is an emerging infection worldwide with predominance in Southeast Asia. The virulent strains K1 and K2 are recognized to cause a new invasive syndrome with metastatic infection [1, 2]. Diabetes mellitus is a significant risk factor for the development of *Klebsiella pneumoniae* liver abscess (KLA). Poor glycemic control results in a worse prognosis and complications including sight-threatening endophthalmitis [3]. We report the first case of KLA in Sri Lanka.

## Case presentation

**Fig. 1:**
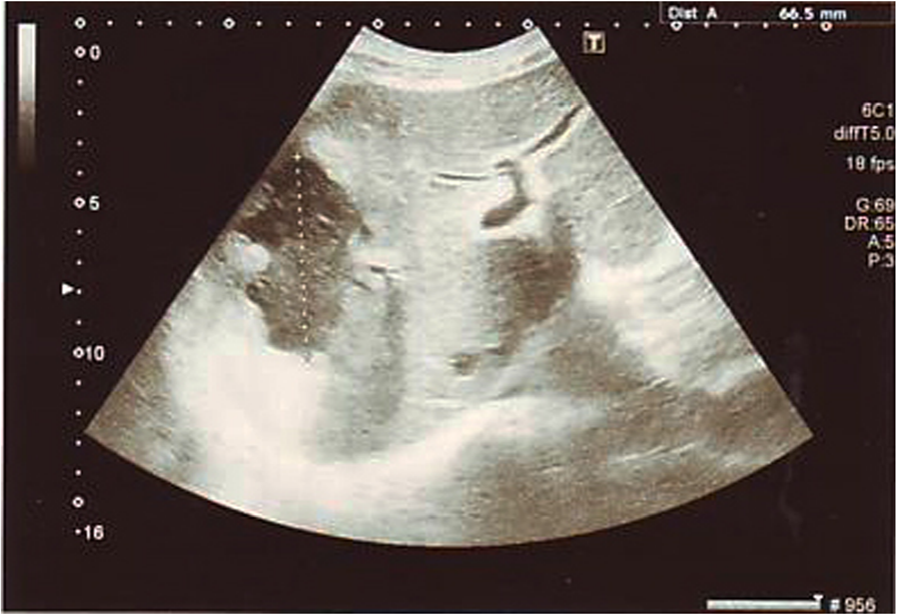
Liver abscess in the right lobe of the liver

A 63-year-old Sinhalese man with diabetes mellitus of 8 years’ duration presented with fever, loose stools, and loss of appetite of 1 week’s duration. He was on diet control for diabetes with poor glycemic control and was not on a proper follow up. On admission he was ill and febrile. An abdominal examination revealed hepatomegaly, 2 cm from right costal margin, which was non-tender. Other systemic examination was unremarkable. An ophthalmoscope examination revealed non-proliferative diabetic retinopathy. A full blood count revealed white cell count of 18 × 10^9^/L with neutrophil predominance. His hemoglobin was 12.2 g/dL and platelet count was 256 × 10^9^/L initially and dropped up to 9.8 g/dl and 63 × 10^9^/L, respectively. Blood film showed severe bacterial infection with sepsis and features suggestive of disseminated intravascular coagulation (DIC). A coagulation profile showed international normalized ratio of 1.7 and activated partial thromboplastin time of 37 seconds. Erythrocyte sedimentation rate was 75 mm/first hour. His C-reactive protein level was 197 mg/dL and his procalcitonin level was 59.9 ng/L. Consecutive blood cultures were positive for *Klebsiella pneumoniae* after 9 and 13 hours. The strain was sensitive to imipenem, meropenem, ceftriaxone, amikacin, and ciprofloxacin. A chest radiograph was normal. Retroviral screening was negative. His fasting blood sugar level was 212 mg/dl. A urine full report showed proteinuria (++) with 3–5 pus cells per high power field and the urine culture was negative. Stool examination was negative for amoebae, ova, or cysts. His initial serum creatinine level was 134 microgram/L and increased up to 647 microgram/L reflecting acute kidney injury. His urine output was satisfactory throughout the course. Serum potassium went up to 6.1 mmol/L and was managed medically. An ultrasound scan of his abdomen revealed a large ill-defined hypoechoic lesion suggestive of a liver abscess in the right lobe of his liver, at segments VII and VIII, measuring 8 × 6 × 5.5 cm in size (Fig. 1). Lower gastrointestinal endoscopy was normal. The abscess was aspirated once by the interventional radiology team under ultrasound guidance yielding 100 ml of thick pus. He was treated with intravenously administered ceftriaxone for 2 weeks and a marked clinical and biochemical improvement was seen. His serum creatinine returned to upper normal limit following resolution of sepsis. A repeat ultrasonography performed at completion of intravenous antibiotic therapy revealed resolving abscess in segment VIII of his liver measuring 5 × 4 × 3.2 cm. As there was considerable clinical and biochemical improvement, he was discharged with a further course of orally administered cefixime and did not require further aspirations.

## Discussion

A liver abscess is a potentially life-threatening infection. Common causes predisposing to occurrence of liver abscess include biliary disease, malignancies of gastrointestinal tract, and pylephlebitis [4]. It is commonly a polymicrobial infection and the common causative organisms well documented in the literature include *Escherichia coli*, streptococci, Enterobacteriaceae, and anaerobes [5–7]. But currently there is an emerging trend of primary liver abscess caused by *Klebsiella* pneumonia.

*Klebsiella pneumoniae* is a Gram-negative bacillus belonging to the genus *Klebsiella*. It is an important nosocomial pathogen and is mostly associated with respiratory and urinary tract infections [8].

Primary liver abscess due to *Klebsiella pneumoniae* was first described in the literature in 1986 in Taiwan [9]. A recent alarming incidence of a new syndrome due to K1 and K2 serotypes of *Klebsiella pneumoniae* is reported, mainly in Taiwan [1, 2]. The cause for the predominance of cases in Southeast Asia is not currently understood and a genetic predisposition is still not proven. However a high fecal carriage of virulent K1 *Klebsiella pneumoniae* ST23 strains was demonstrated in individuals of Southeast Asia and may explain the higher incidence [10]. Intestinal colonization with virulent strains has been shown to be highly associated with development of KLA [11].

This genotype, which is strongly associated with highly invasive disease in Taiwan, has also been reported in three continents denoting its widespread geographic existence [12]. KLA is currently recognized as an emerging infection and an important cause of sight-threatening endophthalmitis in a number of countries worldwide [2, 13].

Patients with diabetes are more prone to develop KLA and related septicemic complications. Also, regardless of etiology, they have longer periods of fever after treatment and prolonged hospital stay [14, 15]. Poor glycemic control impairs the phagocytosis of K1/K2 *Klebsiella pneumoniae*. Thus poorly controlled diabetes increases the susceptibility to K1/K2 *Klebsiella pneumoniae* infections and related liver abscess and complicated endophthalmitis [3]. Also KLA has been shown to be strongly associated with colorectal cancer, particularly in the sigmoid colon and rectum, in the Eastern Asian male population [16].

The *rmpA* gene of *Klebsiella pneumoniae*, Acute Physiologic and Chronic Health Evaluation (APACHE) II score ≥ 2, and septic shock were found to be statistically significant predictors of metastatic infection due to KLA. The predictors of mortality due to KLA include metastatic infection, severity of infection, septic shock, acute respiratory failure, and gas formation in radiography [17]. Molecular analysis and virulence studies have revealed phagocytic resistance associated with K1 serotype and hypervirulence associated with the aerobactin gene [18].

Numerous extrahepatic complications of KLA reported in the literature include meningitis, bacteremia with multiple metastatic abscess formations, epidural abscess formation, necrotizing fasciitis, septic arthritis, and septic pulmonary embolism [19–22]. Of these, sight-threatening endophthalmitis has been commonly reported [23]. Diabetes is a significant risk factor for development of endophthalmitis and predicts a poor visual outcome [24].

Diagnosis of KLA is made based on culture evidence and radiography. On computed tomography the features that suggest KLA include single abscess, unilobar (commonly right) involvement, and solid or multiloculated appearance [25].

Third generation cephalosporins are the preferred choice of treatment in KLA. Pigtail catheter drainage is shown to be protective against metastatic infection and mortality. Treatment with first generation cephalosporins and percutaneous drainage has been found to be associated with low mortality rates, metastatic infection and complications, showing comparable rates to treatment with third generation cephalosporins [17].

In Sri Lanka, one case of a 14-year-old girl has been previously reported with pyogenic liver abscess due to *Klebsiella* species [26]. However, to the best of our knowledge, this is the first reported case of *Klebsiella pneumoniae* causing pyogenic liver abscess in Sri Lanka.

## Conclusion

Considering the reported rise of KLA according to recent literature and its preponderance to occur in Southeast Asian countries, it is worthwhile to suspect *Klebsiella pneumoniae* as an etiological agent in cases of liver abscess in Sri Lanka. Early clinical suspicion mainly in patients with diabetes can lead to early intervention, institution of appropriate therapy, and minimize complications.

## Abbreviations

APACHE: Acute Physiologic and Chronic Health Evaluation
DIC: Disseminated intravascular coagulation
KLA: *Klebsiella pneumoniae* liver abscess

## Authors’ information

WKSK (MBBS, MD, MRCP, FRCP, FCCP), consultant physician; SDNS (MBBS, MD) and JPKJ (MBBS, MD), Senior registrars in Medicine, work at the Teaching Hospital Kandy, Sri Lanka. PNSP is a medical registrar at the Teaching Hospital Kandy, Sri Lanka.
